# Cost effectiveness of adding CMR to evaluation of suspected coronary ischaemia

**DOI:** 10.1186/1532-429X-14-S1-P40

**Published:** 2012-02-01

**Authors:** Deirdre F Waterhouse, Roisin B Morgan, Rory OHanlon

**Affiliations:** 1 Ronan Cahill, CMR Unit Blackrock Clinic, Dublin, Ireland

## Summary

To estimate the cost and diagnostic implications of using CMR alone instead of conventional TTE/TMET work-up to guide patient selection for angiography. Healthcare costs were derived from VHI’s hospital billing system.

## Background

Patient selection for coronary angiography traditionally relies on clinical assessment, treadmill exercise testing (TMET) and transthoracic echocardiography (TTE). Cardiac Magnetic Resonance (CMR) is a relatively novel imaging study, which provides excellent non-invasive assessment of myocardial perfusion and is useful in risk stratification of patients with suspected coronary artery disease (CAD).

## Methods

Consecutive patients referred to our center for CMR evaluation within a 12-month period (january 2010 to january 2011) were enrolled. This population consisted of patients with suspected CAD who were candidates for invasive coronary angiography but first underwent stress cmr for further risk stratification.

## Results

83 Patients (64 male, 19 female) with suspected CAD underwent CMR. 15.4% had ischaemic features on TMET and 47% had evidence of territorial ischaemia on TTE. On CMR evaluation, 38.6% of these were found to have definite CAD. Interestingly, 18% of patients had significant CAD on CMR despite no evidence of ischaemia on TMET and TTE and would not have undergone angiography based on conventional assessment.

In 16 cases (19.3%), planned angiography based on abnormal TTE/TMET was avoided by CMR which excluded a diagnosis of CAD. Furthermore, non-ischaemic causes of cardiac symptoms were discovered on 8.4% of CMR which were undetected on conventional workup.

The use of CMR as first line investigation in assessment of suspected coronary ischaemia would have avoided TMET in 31.3%, TTE in 98.7% and angiography in 25.3%. This would represent a total saving of €18722, or €226 per patient.

## Conclusions

This study demonstrated that a CMR-only approach is the most cost-effective diagnostic strategy for evaluation of CAD. CMR imaging allows accurate selection of patients for invasive management, avoiding unnecessary procedures. CMR was as useful as angiography in guiding revascularisation and is superior to TMET/TTE in detecting ischaemia.

## Funding

None to disclose.

